# The Mental Health in Diabetes Service (MINDS) to enhance psychosocial health: study protocol for a randomized controlled trial

**DOI:** 10.1186/s13063-016-1561-4

**Published:** 2016-09-09

**Authors:** Casey L. O’Brien, Chantal F. Ski, David R. Thompson, Gaye Moore, Serafino Mancuso, Alicia Jenkins, Glenn Ward, Richard J. MacIsaac, Margaret Loh, Simon R. Knowles, Susan L. Rossell, David J. Castle

**Affiliations:** 1 Department of Psychiatry, University of Melbourne, Melbourne, VIC 3010 Australia; 2 Mental Health Service, St. Vincent’s Hospital, Melbourne, VIC 3065 Australia; 3 Centre for the Heart and Mind, Australian Catholic University, Melbourne, VIC 3000 Australia; 4 Department of Endocrinology and Diabetes, St Vincent’s Hospital, Melbourne, VIC 3065 Australia; 5 Department of Medicine, University of Melbourne, Melbourne, VIC 3065 Australia; 6 NHMRC Clinical Trials Centre, University of Sydney, Sydney, NSW 2050 Australia; 7 Department of Psychology, Swinburne University, Melbourne, VIC 3122 Australia

**Keywords:** Diabetes mellitus, Collaborative therapy, Educational, Psychosocial, Randomised controlled trial

## Abstract

**Background:**

After a diagnosis of diabetes mellitus, people not only have to cope with the physical aspects and common complications that require daily self-management, they are also faced with ongoing psychosocial challenges. Subsequently they find themselves having to navigate the health system to engage multidisciplinary supports; the combination of these factors often resulting in reduced health-related quality of life. To maintain optimal diabetes control, interventions need to incorporate psychosocial supports and a skill base for disease management. Therefore, our aim was to evaluate an ‘Optimal Health Program’ that adopts a person-centred approach and engages collaborative therapy to educate and support the psychosocial health of people diagnosed with type I or II diabetes.

**Methods:**

This prospective randomised controlled trial will include 166 people diagnosed with diabetes: 83 in the intervention (Optimal Health Program) and 83 in the control (usual care) group. Participants with type diabetes mellitus will be recruited through hospital outpatient clinics and diabetes community organisations. Participants in the intervention group will receive nine (8 + 1 booster session) sequential sessions, based on a structured treatment manual emphasising educational and psychosocial support self-efficacy and skills building. The primary outcome measures will be generalised self-efficacy (GSE) and health-related quality of life (AQoL-6D and EQ-5D). Secondary measures will be anxiety and depression (HADS), social and workplace functioning (WSAS), diabetes-related quality of life (DQoL), diabetes-related distress (PAID), and type of coping strategies (Brief COPE). In addition, a health economic cost analysis and process evaluations will be performed to assess the economic cost and efficacy of the program’s operations, implementation and service delivery.

**Discussion:**

We envisage that the Optimal Health Program’s emphasis on self-efficacy and self-management will provide participants with the skills and knowledge to achieve increased empowerment and independence in aspects of health, which in turn, will help participants deal more effectively with the physical and psychosocial complexities of diabetes.

**Trial registration:**

ACTRN12614001085662. Registered on 10 October 2014.

## Background

There are an estimated 382 million people globally with diabetes mellitus (DM) − a prevalence of 8.3 % [1]. Far more people are estimated to have blood glucose levels in the prediabetes range, with an increased risk for type 2 diabetes, placing a significant proportion of the world’s population at risk of developing devastating diabetes complications [2]. Diabetes is usually incurable and requires daily and complex self-management; and attention to psychological issues is pivotal to achieving optimum health outcomes [3]. Psychological disorders, including depression, anxiety, maladjustment and eating disorders are highly prevalent in children and adults with DM and are associated with poor prognosis [4–8]. Recognition of such concerns has prompted the incorporation of psychological aspects of DM in national standards of care [4, 9].

At the centre of diabetes care is maintaining optimal glucose target ranges. This is highly reliant on a person’s successful negotiation of healthcare systems and coordination with clinicians [10]. Even when blood glucose levels are within targets, hypoglycaemia can contribute to fear, anxiety and has been associated with decreased quality of life [11]. Emerging evidence suggests that severe nocturnal hypoglycaemia is linked to ‘dead in bed’ syndrome, responsible for approximately 6 % of deaths in people with diabetes aged less than 40 years of age [12]. Understandably, fear of hypoglycaemia is common and difficult to distinguish from anxiety given the shared symptoms e.g., pounding heart, sweating. Improving awareness of anxiety as distinct from hypoglycaemia may encourage persons with diabetes to respond more rapidly, potentially averting a severe episode of illness [11].

Similarly, depression and diabetes have an overlapping relationship, lending further support for holistic programs that facilitate exploration of physical and emotional domains of health. On a biological level, one hypothesis is that depression (which often co-occurs with anxiety) is associated with glucose metabolism via activation of the hypothalamic–pituitary–adrenal cortical (HPA) axis, though the specificity of this link to depression versus other psychiatric conditions is unclear [13]. People who are depressed may also have low motivation and energy, adversely impacting diabetes self-management tasks. Vice versa, if diabetes self-care is challenged, or a person becomes increasingly hopeless about developing complications, a depression presentation can arise. Depression can also present in the context of direct mood effects of chronic hyperglycaemia (high blood sugar).

This evidence highlights the bi-directional relationships between diabetes and mental health, present across the spectrum from mild to more severe clinical presentations. Through supporting emotional wellbeing, it is clear to see how improvements in diabetes can follow, and vice versa, with good support. This randomised controlled trial (RCT) of Mental Health in Diabetes Services Optimal Health Program (MINDS OHP) will adopt a person-centred approach combining collaborative therapy and care coordination to support and improve the psychosocial health of people living with DM.

### Diabetes psychosocial interventions

Diabetes is a challenging condition due to the complex interactions between physiological, psychological and environmental factors and its evolving nature [14, 15]. Recently, interventions and guidelines have minimised advocacy of a hierarchical clinician/patient relationship in favour of a collaborative approach to be delivered by a multidisciplinary team that tailors interventions to each individual’s situation [4, 9, 16]. As such, shared decision making, pragmatic problem solving and promotion of behaviour change strategies are integral to achieving sustainable self-management of DM [4, 5]. Growing evidence has been presented for the use of psychosocial interventions in people with DM, yet questions regarding their efficacy remain [17].

A recent RCT that assessed the effectiveness of cognitive behaviour therapy (CBT) to improve glycaemic control and psychosocial wellbeing in adolescents with type 1 DM (n = 147), demonstrated little improvement in glycaemia [18], but improvements in psychosocial wellbeing (self-efficacy and quality of life) were shown at 3- and 12-month follow-up. Challenges were also noted in the integration of psychosocial services into clinical care, including cost-effectiveness issues, intervention participation, acceptance and attrition [18].

A systematic review and meta-analysis of psychotherapy, antidepressant medication and collaborative care for comorbid DM and depression (14 RCTs; n = 1724) showed that pharmacotherapy and collaborative care reduced depressive symptoms; however, with the exception of sertraline, there was no effect on glycaemic control [5]. The authors concluded that collaborative care containing psychological and pharmacological components is of considerable clinical relevance, although a strong emphasis on DM management and further research into type 1 DM specifically is warranted [5].

A Cochrane review of psychological and pharmacological interventions for depression in DM (eight RCTs; n = 1122) found a moderate and clinically significant effect on depression [6]. Also noted was sparse and inconclusive evidence in relation to the influence of psychological intervention on blood glucose levels, largely due to substantial risk of bias and heterogeneity of populations and interventions [6]. Similar findings regarding CBT and glycaemic control in DM were also reported in another systematic review and meta-analysis (eight studies; n = 1547), which revealed a non-significant trend towards improved blood sugar levels (BSLs) [19].

Recent evidence has established that effective disease management of DM requires strengthening of psychosocial skills, ideally within a collaborative therapy framework integrated with usual medical care [4, 5, 16]. A number of challenges have also been noted including: cost-effectiveness, participation and attrition rates, and delivery that complements and coexists with usual DM health care [6, 17, 18]. As DM has reached epidemic proportions [1]; new and innovative ways of managing it are essential. The MINDS Optimal Health Program (MINDS OHP) adopts a collaborative therapy approach that offers structured yet individualised multidisciplinary support and education to enhance self-efficacy and the psychosocial wellbeing of people with type 1 and type 2 DM. The program recognises that though there are shared psychosocial impacts, type 1 and type 2 diabetes have separate aetiologies and disease processes which will be taken into account in delivering and evaluating the MINDS OHP.

### Translating Research, Integrated Public Health Outcomes and Delivery (TRIPOD)

This RCT is part of a larger research program, TRIPOD, which will evaluate our OHP across three chronic conditions − DM, stroke and chronic kidney disease − inclusive of cost-effectiveness analyses. Based on a collaborative therapy framework [20], the OHP was originally developed to support people with mental illness [21, 22]. An earlier trial of OHP in an Australian adult mental health service demonstrated significant improvements in health and social functioning, a reduction in hospital admissions and net cost savings of AU$6,000 per patient annually [22]. A key aspect of collaborative therapy is recognising that ‘recovery’ and models of chronic health care are not dichotomous [22]. With the intention of enhancing self-efficacy, self-management, care coordination and quality of life, the OHP has been adapted within the broader context of chronic disease. Thus, in the current series of trials our OHP is used to implement this therapeutic framework to enable clinicians and consumers to work systematically towards the achievement of optimal mental health outcomes within mainstream services. The self-management foundations of the OHP are particularly relevant for adults with DM who face the daily challenge of managing often simultaneous aspects of their disease such as diet, exercise, insulin delivery, carbohydrate counting, and blood glucose monitoring, as well as coping with the emotional impact of their condition and care regimen. This protocol describes an RCT (MINDS OHP) that has been designed to evaluate the OHP for people living with DM.

### Pilot study: clarifying the experience of insulin pump users

**Fig. 1:**
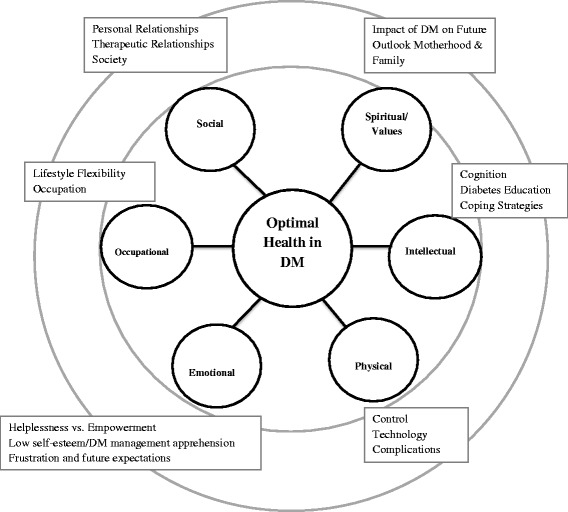
Optimal Health Program (OHP) Optimal Health Wheel (*inner circle*) and diabetes mellitus (DM) pilot study findings (*outer circle*)

Adaptation of the OHP for people with DM was informed by anecdotal evidence, a review of the associated literature, and pilot data. Preliminary work to inform the content and structure of the MINDS manual compromised focus groups of adults with T1DM (two focus groups; n = 3, n = 2, and one individual participant interview) that specifically explored the adaptability of the OHP to DM. The six domains of health from the ‘Optimal Health Wheel’ used in the original OHP were used to guide the direction of discussions (Fig. 1, inner circle). The Framework Method was deemed appropriate to analyse the qualitative content of the focus group data as it provides a systematic model for identifying themes via managing and mapping the data [23]; a specific area of interest was how people dealt with insulin pump therapy [24, 25].

Findings from the focus groups highlighted the importance of self-perception in living and coping with DM. The emotional domain dominated focus group discussions; key themes identified were: a sense of helplessness in gaining control over DM, flexibility of the pump in daily lifestyle; pump malfunction; low self-esteem and self-efficacy regarding adequate management of blood glucose levels; and ongoing concerns about similar ‘out of their control’ experiences in the future. These findings highlight the need for psychosocial supports in the management of DM, especially in regard to self-reflection, problem solving and stress management. Self-efficacy is at the centre of OHP and it is therefore well-placed to support those living with DM. A summary of the findings/themes identified by people with DM associated with each of the Optimal Health Wheel domains are presented in Fig. 1 (see Fig. 1, outer circle).

## Research aims

The aim is to determine whether a DM-specific OHP (MINDS OHP) will improve the psychosocial health of people with diabetes, compared to usual care. The primary objective is to identify the impact of the OHP on levels of self-efficacy and quality of life for those living with DM. Secondary objectives are to evaluate the impact of the OHP on depression, anxiety, social and workplace functioning, diabetes-related distress, illness perceptions of DM, and coping with DM.

In addition, a health economic cost analysis will be performed, assuming an Australia-wide implementation, to identify any cost savings of the MINDS OHP intervention over current practice. Quality-adjusted life years (QALYs) will be measured using the Assessment of Quality of Life-6D (AQoL-6D) [26] and European Quality of Life-5 dimensions (EQ-5D) [27]. Process evaluation including focus group interviews will also be conducted with participants with diabetes and diabetes clinicians to assess the effectiveness of the MINDS OHP operations, implementation, and service delivery.

## Methods

### General design

This is a prospective randomised controlled trial examining the effectiveness of the OHP specifically adapted for adults with DM, focused on improving participants’ psychosocial health.

The intervention period lasts for 8 weeks with an additional booster session, and is compared with usual care. The study protocol and its amendments were approved by the Human Research Ethics Committee of St Vincent’s Hospital Melbourne (decision number 036/14, 21 April 2015). An executive steering committee (all authors) is responsible for study planning, conduct and monitoring.

### Setting

The study will be conducted at St Vincent’s Hospital, a large metropolitan teaching hospital in Melbourne, Australia. As of March 2015 the endocrinology and diabetes unit had over 1000 people with diabetes enrolled in their patient database: 370 people with type I DM and 1313 with type 2 DM. The necessary volume of clinical cases and expertise required for this study is well established in this unit. As a focus of the OHP is care co-ordination, the program has been integrated into existing services through transparent, planned and close collaboration with the diabetes unit, i.e. diabetes educators, endocrinologists, social workers, and dieticians.

### Participants

A total of 166 participants with a diagnosis of DM will be recruited into the RCT. Inclusion criteria are: (1) have a DM diagnosis, confirmed by medical records; (2) be aged 18 years or above; and (3) be able to converse in English without an interpreter. Exclusion criteria are: (1) presence of developmental disability or amnestic syndrome impairing their ability to learn from the intervention; and (2) comorbid serious acute medical illness defined by the treating physician. As the OHP adopts a holistic approach to managing chronic disease, patients may enter the program at any stage along the continuum of care. De-identified records will be collected on how many people with diabetes were approached or self-referred to the study including reasons for decline, to assess for potential selection bias.

Power was calculated to detect a medium effect size of d = 0.50. This was chosen as a clinically meaningful effect size that may be compared with previous RCT research in the area of chronic disease management programs [28]. The calculations assumed two primary outcomes (health-related quality of life and General Self-efficacy Scale (GSE) scores), four assessment points (baseline, 3-month, 6-month, and 12-month follow-ups), a study-wide type I error rate (α) of .05, and hence a type II error rate (β) of 0.20 (power of 0.80), a correlation of post-treatment scores with baseline measurements (ρ) of 0.81, and a two-tailed statistical test [29]. To detect the effect size of d = 0.50, 66 participants in each of the control and intervention groups will be required. Allowing for up to 20 % attrition, a total of 166 participants, or 83 in each group will be recruited.

### Study procedures

#### Consent

The process of consent will be in accordance with the Declaration of Helsinki. All eligible people with diabetes will be fully informed that they are being asked to participate in an RCT. The procedures involved in the study, and the chances of being assigned randomly to one of two groups will be explained verbally and via an information sheet approved by St Vincent’s Hospital Human Research Ethics Committee. A signed consent form will be obtained from each participant. Participants will be made aware of their right to withdraw from the study at any time without any effects on their usual clinical management.

#### Randomisation and blinding

Using a computer-generated block randomisation sequence created by a person not directly involved in the study, participants will be allocated to treatment or control group. The allocation sequence will be generated using random numbers. Participants will be randomised progressively as they consent. Due to the nature and length of the intervention, it is not possible to mask either participant or investigator to the treatment allocation.

#### Recruitment

**Fig. 2:**
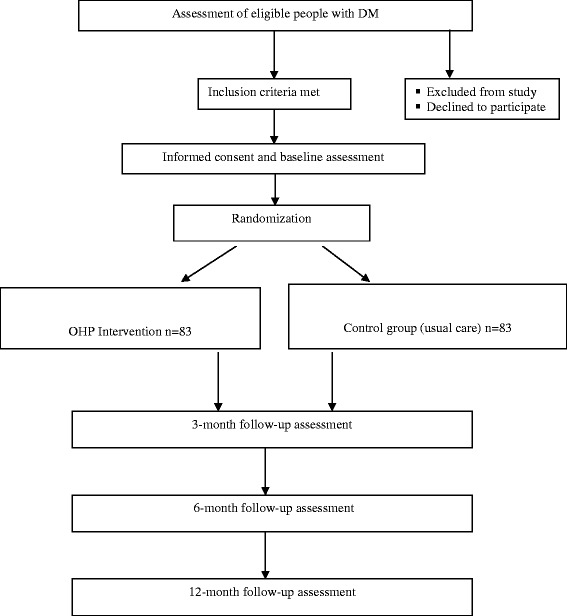
Flowchart of the Mental Health in Diabetes Services Optimal Health Program (MINDS OHP) randomised controlled trial (RCT)

Potential participants with DM at St Vincent’s Hospital will be identified by the diabetes clinical staff (e.g. endocrinologist, diabetes educator) and provided with a study flyer. People with diabetes will be asked permission for a researcher to approach them to discuss the program in more detail. If agreeable, people with diabetes will be approached, informed and formally consented by the research assistant. Study flyers will also be posted online through community organisations and will include contact details for the research team. Participants from the community may contact researchers directly to request further information. Recruitment will occur over an 18-month period (see Fig. 2).

### Intervention: MINDS OHP

The MINDS OHP is delivered in nine (8 + 1 booster session) sequential sessions based on a structured treatment manual Optimal Health Program: My Workbook (version 3) [30]. The sessions will be one-on-one, delivered face-to-face, and participants in rural and regional areas will have the option of participating in sessions via videoconferencing or telephone. Participants are encouraged throughout the program to identify which areas of DM other health domains upon which they would like to focus. Sessions are approximately 1 hour in duration and held weekly, apart from the ‘booster’ session, which is held 3 months after session 8. Learning is cumulative with each session designed to build on the previous session including tasks to complete between sessions, such as journaling and sleep logs.

In summary, session 1 introduces the OHP within the six domains of the Optimal Health Wheel: social, physical, emotional, intellectual, employment (engagement) and spiritual/values. This session provides participants with the opportunity to explore and understand their DM and other health priorities from a holistic perspective. Sessions 2 and 3 initiate development of a health plan exploring the implications and potential complications of DM in terms of strengths and vulnerabilities. In session 3, further understanding is developed around the effects of stress or blood glucose levels with strategies to mitigate these stressors and informing their health plans. Session 4 focuses on metabolic monitoring and medication management (e.g. use of insulin pumps). Session 5 expands the health plan to include key DM partnerships and supports in the community and online, for example Diabetes Australia [31], and HypoActive [32]. Change enhancement is the focus in session 6, in terms of understanding past events and establishing new proactive avenues for change. The aim of session 7 is goal setting via creative problem solving and planning around the complexities of DM. Session 8 strategises DM health and emergency care planning that incorporates wellbeing maintenance and sustainability. The goal of the ‘booster session’ (session 9) is to review health plans, consolidate progress, and reflect on achievements towards health-related goals.

A facilitator with a health background (e.g., nurse, psychologist) will conduct each session. All facilitators will complete a 2-day workshop plus regular supervision and fidelity checks. Facilitators will draw on DM-specific information in concordance with individual circumstances. Examples include the relationship between stress and hypoglycaemia, availability of DM supports in the community, and coping strategies for addressing anxiety related to changes in BSLs. The emphasis is on collaboration between facilitator and participant to arrive at program goals that stem from the participant’s main concerns and needs. The facilitator will encourage participants to identify their early warning signs of stress and illness and integrate healthy coping strategies to prevent stress build-up. Facilitators may also discuss and arrange referrals for other services in conjunction with the multidisciplinary team depending on participant needs. Additionally, facilitators may work with the multidisciplinary team to coordinate visits with other hospital appointments.

### Control

The comparison group will receive usual care and no MINDS OHP intervention. As participants will be recruited from a variety of settings (hospital outpatients, community organisations) we anticipate variation in standard care received. To capture this variation, all participants will complete the Health Care Utilisation Questionnaire (HCUQ) [33] at each time point. Participants in the control group will have the option of completing the MINDS OHP at the end of the trial once evaluation is complete.

### Outcome measurements

Table 1 displays primary and secondary outcome measures and time points. Primary measures are changes in: health-related quality of life as assessed by the AQoL-6D [26], consisting of six separately scored dimensions of good health and a simple global ‘utility’ score and EQ-5D [27]; and self-efficacy as measured by the General Self-Efficacy Scale (GSE) [34], a general sense of perceived self-efficacy in regarding daily hassles as well as adaptation to stressful life events. Secondary measures are: the Diabetes Quality of Life (DQoL) [35] measure, a screen for problem or risky substance use in adults as assessed by the Alcohol, Smoking and Substance Involvement Screening Test (ASSIST) [36] developed by the World Health Organization (WHO); coping strategies as measured by an abbreviated version of the COPE inventory [37], the Brief COPE [38]; treatment expectancy and rationale credibility in clinical studies as assessed by the Credibility/Expectancy Questionnaire (CEQ) [39]; clinical indices such as body mass index (BMI) and glycated haemoglobin (HbA1c); diabetes-related psychosocial self-efficacy measured by the Diabetes Empowerment Scale (DES) [40]; symptom severity and caseness (number meeting clinical disorder threshold) of anxiety and depression as assessed by the Hospital Anxiety and Depression Scale (HADS) [41]; health care utilisation for economic evaluation purposes assessed by the Health Care Utilisation Questionnaire (HCUQ) [32]; diabetes-related emotional distress measured by the Problem Areas in Diabetes Scale (PAID) [42]; perceived acceptability of treatment assessed using the Treatment Evaluation Inventory-Short Form (TEI-SF) [43]; a 10-item measure of the Big Five Inventory (BFI) personality dimensions [44]; and impact of a person’s mental health difficulties on their ability to function via the Work and Social Adjustment Scale (WSAS) [45].

**Table 1:** Primary and secondary outcome assessments and time points for MINDS

Assessment tools	Baseline	3-month	6-month	12-month
Primary outcomes				
AQoL-6D (20 items)	X	X	X	X
GSE (10 items)	X	X	X	X
Secondary outcomes				
DQoL (15 items)	X	X	X	X
ASSIST (6 items)	X	X	X	X
Brief COPE (28 items)	X	X	X	X
CEQ (6 items)	X			
Clinical Indices (e.g. BMI)	X	X	X	X
DES (8 items)	X	X	X	X
EQ-5D-3L (6 items)	X	X	X	X
HADS (14 items)	X	X	X	X
HCUQ (17 items)	X	X	X	X
PAID (20 items)	X	X	X	X
TEI-SF (9 Items)		X		
BFI-10 (10 items)	X			
WSAS (5 items)	X	X	X	X

*AQoL-6D* Assessment of Quality of Life-6 Dimensions [26], *GSE* General Self-efficacy Scale [34], *DQoL* Diabetes Quality of Life [35], *ASSIT* Alcohol, Smoking and Substance Involvement Screening Test [36], *Brief COPE* abbreviated version of the COPE Inventory [38], *CEQ* Credibility/Expectancy Questionnaire [39], *BMI* body mass index, *DES* Diabetes Empowerment Scale [40], *EQ-5D-3L* European Quality of Life-5 Dimensions-3 Levels [27], *HADS* Hospital Anxiety and Depression Scale [41], *HCUQ* Health Care Utilisation Questionnaire [33], *PAID* Problem Areas in Diabetes Scale [42], *TEI-SF* Treatment Evaluation Inventory-Short Form [43], *BFI-10* Big Five Inventory-10 item [44], *WSAS* Work and Social Adjustment Scale [45]

Due to variability of usual care across all participants, key aspects of usual care will be assessed via responses on the HCUQ [32]. If possible, medical records will be reviewed to ascertain DM diagnostic information and clinical indicators such as HbA1c.

### Program assessment and treatment fidelity

The OHP facilitators will receive training, a structured manual/protocol, and monthly group supervision with the clinical investigators (with individual supervision provided as needed in between group sessions). The purpose of supervision will be to discuss problems in study procedures and ensure standardised activity. The OHP sessions will be audio recorded with a random selection rated by independent assessors according to compliance with OHP protocols. Identified variations from protocols will be communicated back to the facilitator. In addition, content of sessions including participant needs and concerns will be raised at supervision meetings. All facilitators will complete a summary of each session using a standard template and send these notes to the research team. Session notes will include OHP topics covered, participant concerns raised, and needs for supervision.

Post-intervention focus groups will be held for clinicians and for participants from the control and intervention groups. Participants will be informed that the purpose of the focus group is to gain an in-depth understanding of their experiences of the study, advantages and disadvantages of conducting the study/program in their services (for service providers), and suggestions for components to include or exclude from the OHP. To increase objectivity, focus group facilitators will be independent researchers who were not OHP facilitators.

### Statistical analyses

Intention-to-treat analyses will be employed to prevent overestimation of efficacy. Categorical variables will be analysed using chi-squared tests (or Fisher’s exact test for small samples). A mixed-effects model, repeated measures (MMRM) approach will be used to examine the longitudinal profile of all continuous variables at 3, 6 and 12 months post-baseline. For all MMRM analyses, baseline scores will be used as covariates and the models will include prespecified fixed effects of treatment, clinician, and time, and treatment-by-time and treatment-by-clinician interactions.

Secondary analyses using analysis of covariance will be conducted to compare change scores during treatment and follow-up phases for all primary, secondary, and process outcomes using the fixed, continuous covariate of baseline score and time from diagnosis as well as the categorical fixed effects of treatment group, clinician, and treatment-by-clinician interactions. Sub-analyses will be conducted on participants with T1 and T2DM as these have different disease processes and aetiologies.

Although the attrition rate is not expected to vary by treatment condition, we will attempt to identify key predictors of attrition status (i.e. demographic and baseline clinical characteristics) and test for differences between conditions. Assuming the data are missing at random, several procedures offer effective approaches that may attenuate attrition. Maximum likelihood models (i.e. MMRM), with time as a random variable, allow the use of all available data from all assessments, reducing bias and increasing power [46]. In addition, multiple imputation procedures that utilise the expectation-maximization (EM) algorithm with bootstrap estimates of standard errors will be used to address attrition. The application of these procedures can provide unbiased estimates, even in the face of substantial missing data [47].

A full economic evaluation will occur alongside the proposed RCT. Healthcare outcomes and costs will be compared between participants in control and interventional conditions. Healthcare system (medical record) and self-reported information via the HCUQ [32] will be used to generate analyses. The utility measurements of participant quality of life will be assessed using AQoL-6D [26] developed in Australia and the EQ-5D-3L [40] developed in Europe. The potential long-term (lifetime) impact on cost and effectiveness of intervention beyond the trial period will be extrapolated using the Markov process modelling method.

## Discussion

Diabetes is a multifaceted chronic disease that places many at high risk of developing diabetes-related complications and conditions when management is not within optimal target ranges [2]. The complexity of DM increases due to the impact on psychosocial health and the necessity of navigating the health system for not only physical but also psychological purposes [3–8]. To our knowledge, this will be the first trial to test a collaborative therapy OHP integrated in the clinical setting that focuses on the psychosocial health of people living with DM. We investigate in our trial the effects of an 8-week psychosocial intervention to improve self-efficacy, self-management and quality of life of people living with DM.

The MINDS OHP has several strengths, primarily the recognition for coordinated care aimed at enhancing the wellbeing of people experiencing chronic disease. The emphasis on self-efficacy and self-management fosters health promotion as it provides the patient with the basic building blocks to take control of their illness and recovery. By working towards this shift in focus of the person’s illness from being ‘dependent on’ services to being ‘supported by’ services, the MINDS OHP provides enablers for increased independence and empowerment.

If the findings are positive, we envisage that the OHP will be translated into the hospital’s clinical pathway for referral and engagement across all chronic diseases, as well as included as a core component of educational training accessible to all staff. In addition the quality control component of this trial, via process evaluation, will offer further insight into how the intervention can best be adapted and integrated into the general medical or community setting.

We believe that this innovative trial will contribute to the knowledge of interventions aimed at supporting people with DM and broaden the focus from symptoms to include psychosocial factors such self-efficacy, wellbeing and community supports.

### Trial status

Patient recruitment commenced April 2015 and data collection will continue until at least December 2017. ANZCTR no. 12614001085662

## Abbreviations

AQoL-6D: Assessment of quality of Life-6 dimensions
ASSIST: Alcohol, smoking and substance involvement screening test
BFI-10: Big five inventory-10 item
BMI: Body mass index
BRIEF COPE: Abbreviated version of the COPE inventory
BSLS: Blood sugar levels
CBT: Cognitive behaviour therapy
CEQ: Credibility/expectancy questionnaire
DES: Diabetes empowerment scale
DM: Diabetes mellitus
DQoL: Diabetes quality of life brief clinical inventory
EM: Expectation-maximization
EQ-5D-3L: European quality of life-5 dimensions-3 levels
GSE: General self-efficacy scale
HADS: Hospital anxiety and depression scale
HbA1c: Glycated haemoglobin
HCUQ: Health care utilisation questionnaire
MMRM: Mixed-effects model, repeated measures
OHP: Optimal health program
PAID: Problem areas in diabetes scale
QALY: Quality-adjusted life year
RCT: Randomised controlled trial
TEI-SF: Treatment evaluation inventory-short form
TRIPOD: Translating research, integrated public health outcomes and delivery
WSAS: Work and social adjustment scale
